# Patient‐reported quality of life in adolescents and young adults with cancer who received radiation therapy

**DOI:** 10.1002/cam4.6082

**Published:** 2023-05-18

**Authors:** Kelsey L. Corrigan, Bryce B. Reeve, John M. Salsman, Elizabeth J. Siembida, Grace L. Smith, Maria C. Swartz, Kamaria L. Lee, Faraz Afridi, Lauren M. Andring, Andrew J. Bishop, Jillian R. Gunther, J. Andrew Livingston, Susan K. Peterson, Michael Roth

**Affiliations:** ^1^ Department of Radiation Oncology The University of Texas MD Anderson Cancer Center Houston Texas USA; ^2^ Department of Population Health Sciences, Department of Pediatrics, Center for Health Measurement Duke University School of Medicine Durham North Carolina USA; ^3^ Department of Social Sciences and Health Policy Wake Forest University Winston‐Salem North Carolina USA; ^4^ Institution of Health System Science, Northwell Health Manhasset New York USA; ^5^ Department of Pediatrics The University of Texas MD Anderson Cancer Center Houston Texas USA; ^6^ Department of Sarcoma Medical Oncology The University of Texas MD Anderson Cancer Center Houston Texas USA; ^7^ Department of Behavioral Science The University of Texas MD Anderson Cancer Center Houston Texas USA

**Keywords:** adolescents and young adults, cancer, health‐related quality of life, patient‐reported outcomes, radiation therapy

## Abstract

**Background:**

Radiation therapy (RT) is a common treatment for adolescents and young adults (AYAs, 15–39 years old) with cancer; however, it may cause toxicities that affect health‐related quality‐of‐life (HRQOL). Thus, we assessed HRQOL in AYAs before, during, and after RT.

**Methods:**

We identified 265 AYAs who completed HRQOL PROMIS® surveys before (*n* = 87), during (*n* = 84), or after (*n* = 94) RT. Higher PROMIS® score represents more of the concept. Mean scores were compared to the general US population and minimally important differences (MIDs) were used to evaluate the impact of cancer on HRQOL. Linear regression modeling was used to evaluate the effect of clinical and demographic factors on PROMIS scores.

**Results:**

Median [IQR] age was 26 [20–31] years. Cancer types varied; most had sarcoma (26%) or CNS malignancy (23%). Compared to the general US population, the before RT cohort had worse anxiety (mean score 55.2 vs. 50, MID 3, *p* < 0.001) and the during RT cohort had worse global physical health (mean score 44.9 vs. 50, MID 5, *p* < 0.001). In the during RT cohort, patients with regional/distant disease had significantly worse pain (*B* = 15.94, *p* < 0.01) and fatigue (*B* = 14.20, *p* = 0.01) than patients with localized disease. In the after RT cohort, adolescents (15–18 years) and young adults (26–39 years) had worse global physical health (*B* = ‐6.87, *p* < 0.01, and *B* = ‐7.87, *p* < 0.01, respectively) and global mental health (*B* = ‐6.74, *p* < 0.01, and *B* = ‐5.67, *p* = 0.01, respectively) than emerging adults (19–25 years).

**Conclusions:**

AYAs with cancer receiving RT experience impairments in various domains of HRQOL. Advanced cancer stage may contribute to poorer short‐term HRQOL and developmental stage may contribute to differing long‐term HRQOL.

## 1 INTRODUCTION

Approximately, 89,000 adolescents and young adults (AYAs, age: 15–39 years old) are diagnosed with cancer each year in the United States and treated with a combination of surgery, systemic therapy, and/or radiation therapy (RT).^1^ Toxicities from these treatments may negatively impact AYA health‐related quality of life (HRQOL) during treatment and/or for many decades posttreatment. The primary method for assessing cancer patient HRQOL is through patient‐reported outcome (PRO) measures, which have been shown to be a more accurate representation of HRQOL than provider‐reported outcomes and are being increasingly implemented into cancer studies.^2^, ^3^, ^4^, ^5^ In the AYA population, PROs are especially useful for investigating HRQOL as this group has different treatment tolerability and unique psychosocial needs compared with pediatric and older adult cancer populations.^6^

As various modalities are used to treat cancer, PROs may help distinguish between the impact of different cancer treatments on HRQOL in order to direct appropriate resources to patients undergoing specific treatments. For example, the field size, dose, and modality used to deliver RT creates a toxicity and HRQOL profile that differs from the systemic effects of chemotherapy. However, limited data have been published reporting these outcomes in AYAs receiving RT. A recent scoping literature review found only six studies that analyzed patient‐reported toxicity and/or HRQOL in AYAs with cancer receiving RT, with only one study^7^ publishing in‐depth information on radiation technique, intent, dose, and fractionation.^8^ None of the studies specifically examined HRQOL in AYAs during or shortly after RT; consequently, the impact of active RT on AYA HRQOL is not known.

Our institution has a dedicated AYA clinic which provides comprehensive care to AYAs undergoing active cancer treatment or in survivorship. In order to assess HRQOL before, during, and after RT, we performed a cross‐sectional study of patients from our AYA clinic who completed a PRO survey assessing various aspects of HRQOL. Our objective was to describe the landscape of HRQOL in AYAs undergoing RT across cancer sites and to identify specific subpopulations that may be at‐risk for poor short‐ and long‐term HRQOL.

## 2 METHODS

### 2.1 Patient selection

For this retrospective cross‐sectional study, we included AYAs, aged 15–39 years at cancer diagnosis, who received RT and self‐completed a PRO inventory between July 2019 and February 2022 at the University of Texas MD Anderson Cancer Center AYA clinic. Each patient included for analysis completed the survey once and was stratified into one of three cohorts based on the date of survey completion in relation to their RT: before RT, during RT (during RT or within 2 months of RT completion), or after RT (2 months or greater following RT completion). Patient demographic and clinical variables were extracted from the electronic medical record (EMR). Developmental stage was defined as: 15–18 years old “adolescents” versus 19–25 years old “emerging adults” versus 26–39 years old “young adults” at the time of survey completion. TNM classifications were not collected as they vary between and within each cancer site, and we believed it would not provide useful information due to the heterogeneity of our cohort which is made up of many cancer types. This study was approved by the Institutional Review Board at the University of Texas MD Anderson Cancer Center.

### 2.2 Measures

We used Patient‐Reported Outcomes Measurement Instrument System® (PROMIS®) instruments to evaluate HRQOL in AYAs with cancer in our study according to the recently published National Clinical Trials Network AYA PRO Task Force core battery, which recommends using patient‐reported PROMIS instruments to evaluate HRQOL in AYAs with cancer.^9^ AYA patients were asked to self‐complete the PROMIS inventory electronically at their household via a message sent from the EMR, or via paper‐and‐pencil in clinic. The inventory contained nine validated instruments from the adult, English versions of PROMIS: v1.2 Global Physical Health, v1.2 Global Mental Health, v2.0 Ability to Participate in Social Roles and Activities (Social Roles) Short Form 4a, v2.0 Cognitive Function Short Form 4a, v1.0 Pain Interference (Pain) Short Form 4a, v1.0 Fatigue Short Form 4a, v1.0 Sleep Disturbance Short Form 4a, v1.0 Anxiety Short Form 4a, and v1.0 Depression Short Form 4a.^10^, ^11^

The adult PROMIS profile scoring tables were used to convert the raw sum score to a T‐score for each measure. A T‐score is a standardized score that represents the relative position of a patient's score on a PROMIS measure as compared to the reference general US population. A T‐score of 50 represented the mean from the general US population; with the exception of Sleep Disturbance where the mean of 50 is a mix of clinical and general US population samples.^12^, ^13^ Patient T‐scores were omitted if ≥25% of individual items within a short form were missing. For global physical health, global mental health, social roles, and cognitive function measures, higher scores represented better functioning. For pain, fatigue, sleep disturbance, anxiety, and depression measures, higher scores represented a greater symptom burden.

Minimally important differences (MIDs), which are the smallest differences in PRO scores that carry implications for patient care,^14^ have been previously established in cancer patients for some of the PROMIS HRQOL domains: Pain (4.0–6.0 points), fatigue (2.5–5.0 points), anxiety (3.0–4.5 points), and depression (3.0–4.5 points).^15^ For PROMIS HRQOL domains without established MIDs in cancer patients (global physical health, global mental health, social roles, cognitive function, and sleep disturbance), we used one half of the standard deviation (SD) as the MID (5.0 points), which has been similarly performed in prior studies utilizing this strategy.^16^, ^17^

Score thresholds for mild or worse severity have been established using PROMIS score cut points for the following domains: Social roles (threshold: 45), cognitive function (threshold: 45), pain (threshold: 55), fatigue (threshold: 55), sleep disturbance (threshold: 55), anxiety (threshold: 55), and depression (threshold: 55).^18^ Score thresholds for fair or poor severity have been established using PROMIS score cut points for the following domains: Global physical health (threshold: 42), and global mental health (threshold: 40).^18^

### 2.3 Statistical analysis

This study analyzed each PROMIS measure within the three cohorts separately: before, during, and after RT. Mean scores with SD were calculated for each measure and compared to the mean score from the general US population (mean of 50). For each domain, if the mean score indicated worse function as compared to the general population and exceeded the lower bound of the MID, thus indicating the score was different enough that may cause a difference in patient value or care, then it was considered to be meaningfully impacted by cancer. For these mean scores that met the MID, one‐sample t‐tests were used to ensure a statistically significant difference between the study sample PROMIS mean and the US general population mean. The distribution of PROMIS scores were modeled on violin plots for each domain within the three cohorts. The percentage of scores that were worse than the threshold for mild (social roles, cognitive function, pain, fatigue, sleep disturbance, anxiety, and depression) or fair (global physical health, global mental health) severity for each domain were calculated and described. Multivariable linear regression modeling was used to evaluate the effect of RT cohort on PROMIS scores within the entire population and the effect of covariates on PROMIS scores within each cohort. Univariate modeling of the following variables was used to identify predictors of worse PROMIS scores for each model: sex, race and ethnicity, developmental stage, cancer type, disease stage, concurrent treatment with systemic therapy and RT (CRT), and RT cohort. All covariates except some cancer types had *p*‐value less than 0.25 on univariate testing and were included in the final multivariable regression models. All cancer types were included in the final regression models due to the clinically significant differences between cancer types. Linear regression estimated parameters (*B*, representing the relationship between predictor variables and PROMIS score) were reported and multivariable linear relationships with *p*‐values ≤ 0.01 were considered statistically significant. Significant relationships between covariates and PROMIS scores were deemed clinically meaningful if the difference in PROMIS score means between covariate groups met the MID as described above. Statistical analyses were conducted using IBM SPSS, version 22.0. Figures were generated using GraphPad Prism, Version 9.2.0.

## 3 RESULTS

### 3.1 Patient and clinical characteristics

Two hundred sixty‐five AYAs who received RT completed HRQOL surveys and were included in the analysis. The median (interquartile range [IQR]) age of all patients was 26 (20–31) years at the time of survey completion. Demographic and clinical characteristics for included AYAs are described in Table 1. Histopathologic designations for each cancer type among cohorts is described in Table S1.

**TABLE 1:** Patient demographic and clinical characteristics (*n* = 265).

Characteristics	Before RT (*n* = 87)	During RT^a^ (*n* = 84)	After RT^b^ (*n* = 94)	Total (*n* = 265)
	*N* (%)	*N* (%)	*N* (%)	*N* (%)
Median (IQR) age (years)	28 (21–33)	26 (20–32)	23 (20–27)	26 (20–31)
Age at survey completion (years)				
15–18 (Adolescents)	9 (10.3%)	12 (14.3%)	30 (31.9%)	51 (19.2%)
19–25 (Emerging adults)	30 (34.5%)	25 (29.8%)	32 (34.0%)	87 (32.8%)
26–39 (Young adults)	48 (55.2%)	47 (56%)	32 (34.0%)	127 (47.9%)
Sex				
Female	45 (51.7%)	32 (38.1%)	48 (51.1%)	125 (47.2%)
Male	42 (48.3%)	52 (61.9%)	46 (48.9%)	140 (52.8%)
Race and ethnicity				
Non‐Hispanic white	57 (65.5%)	46 (54.8%)	51 (54.3%)	154 (58.1%)
Hispanic or non‐white^c^	30 (34.5%)	38 (45.2%)	43 (41.9%)	111 (41.9%)
Cancer type				
Breast	12 (13.8%)	6 (7.1%)	7 (7.4%)	25 (9.4%)
Central nervous system	14 (16.1%)	27 (32.1%)	19 (20.2%)	60 (22.6%)
Gastrointestinal	5 (5.7%)	3 (3.6%)	3 (3.2%)	11 (4.2%)
Genitourinary	1 (1.1%)	1 (1.2%)	2 (2.1%)	4 (1.5%)
Gynecologic	1 (1.1%)	0 (0.0%)	2 (2.1%)	3 (1.1%)
Head and neck	5 (5.7%)	9 (10.7%)	14 (14.9%)	28 (10.6%)
Leukemia	3 (3.4%)	3 (3.6%)	4 (4.4%)	10 (3.8%)
Lymphoma	17 (19.5%)	7 (8.3%)	15 (16%)	39 (14.7%)
Lung	1 (1.1%)	4 (4.8%)	2 (2.1%)	7 (2.6%)
Melanoma/skin	4 (4.6%)	3 (3.6%)	2 (2.1%)	9 (3.4%)
Soft tissue sarcoma	24 (27.6%)	21 (25.0%)	24 (25.5%)	69 (26.0%)
Disease stage at radiation treatment				
Local	36 (41.4%)	53 (63.1%)	52 (55.3%)	141 (53.2%)
Regional	29 (33.3%)	17 (20.2%)	23 (24.5%)	69 (26.0%)
Distant	22 (25.3%)	14 (16.7%)	19 (20.2%)	55 (20.8%)
RT intent				
Definitive or consolidative	28 (32.2%)	21 (25.0%)	34 (36.2%)	83 (31.3%)
Preoperative	6 (6.9%)	5 (5.9%)	8 (8.5%)	19 (7.2%)
Postoperative	37 (42.5%)	42 (50.0%)	31 (33.0%)	110 (41.5%)
Palliative	16 (18.4%)	16 (19.1%)	21 (22.3%)	53 (20.0%)
Median (IQR) total radiation dose (Gy)	48 (30–57)	50.4 (40–60)	50.4 (30–60)	50 (30–60)
Median (IQR) number of fractions	20 (12–30)	25 (15–30)	28 (16–30)	25 (15–30)
RT modality and technique				
2D/3D RT	20 (23.0%)	24 (28.6%)	18 (19.1%)	48 (18.1%)
IMRT/VMAT	45 (51.7%)	44 (52.3%)	64 (68.1%)	153 (57.7%)
PSPT	10 (11.5%)	11 (13.1%)	3 (3.2%)	28 (10.6%)
IMPT	6 (6.9%)	10 (11.9%)	5 (5.3%)	21 (7.9%)
Electron therapy	0	0	2 (2.1%)	2 (0.8%)
SRS	2 (2.2%)	1 (1.2%)	1 (1.1%)	4 (1.5%)
SBRT	4 (4.6%)	4 (4.8%)	1 (1.1%)	9 (3.4%)
Receipt of surgery				
No	38 (43.7%)	33 (39.3%)	40 (42.6%)	111 (41.9%)
Yes	49 (56.3%)	51 (60.7%)	54 (57.4%)	154 (58.1%)
Receipt of neoadjuvant systemic therapy				
No	49 (56.3%)	57 (67.9%)	52 (55.3%)	158 (59.6%)
Yes	38 (43.7%)	27 (32.1%)	42 (44.7%)	107 (40.4%)
Receipt of concurrent systemic therapy				
No	77 (88.5%)	66 (78.6%)	66 (70.2%)	209 (78.9%)
Yes; cisplatin	5 (5.7%)	10 (11.9%)	17 (18.1%)	32 (12.1%)
Yes; vincristine/ifosfamide	0	2 (2.4%)	4 (4.3%)	6 (2.3%)
Yes; temozolomide	5 (5.7%)	9 (10.7%)	7 (7.4%)	21 (7.9%)
Receipt of adjuvant systemic therapy				
No	45 (51.7%)	34 (40.5%)	46 (48.9%)	125 (47.2%)
Yes	42 (48.3%)	50 (59.5%)	48 (51.1%)	140 (52.8%)

Abbreviations: IMPT, intensity‐modulated proton therapy; IMRT, intensity‐modulated radiation therapy; IQR, interquartile range; PSPT, passive scatter proton therapy; RT, radiation therapy; SBRT, stereotactic body radiation therapy; SRS, stereotactic radiosurgery; VMAT, volumetric modulated arc therapy.

^a^ Survey completed during RT or within 2 months following completion of RT.

^b^ Survey completed 2 months or greater following completion of RT.

^c^ Non‐White includes Black, Asian, Native American, other, and no response.

### 3.2 Before RT cohort

**FIGURE 1:**
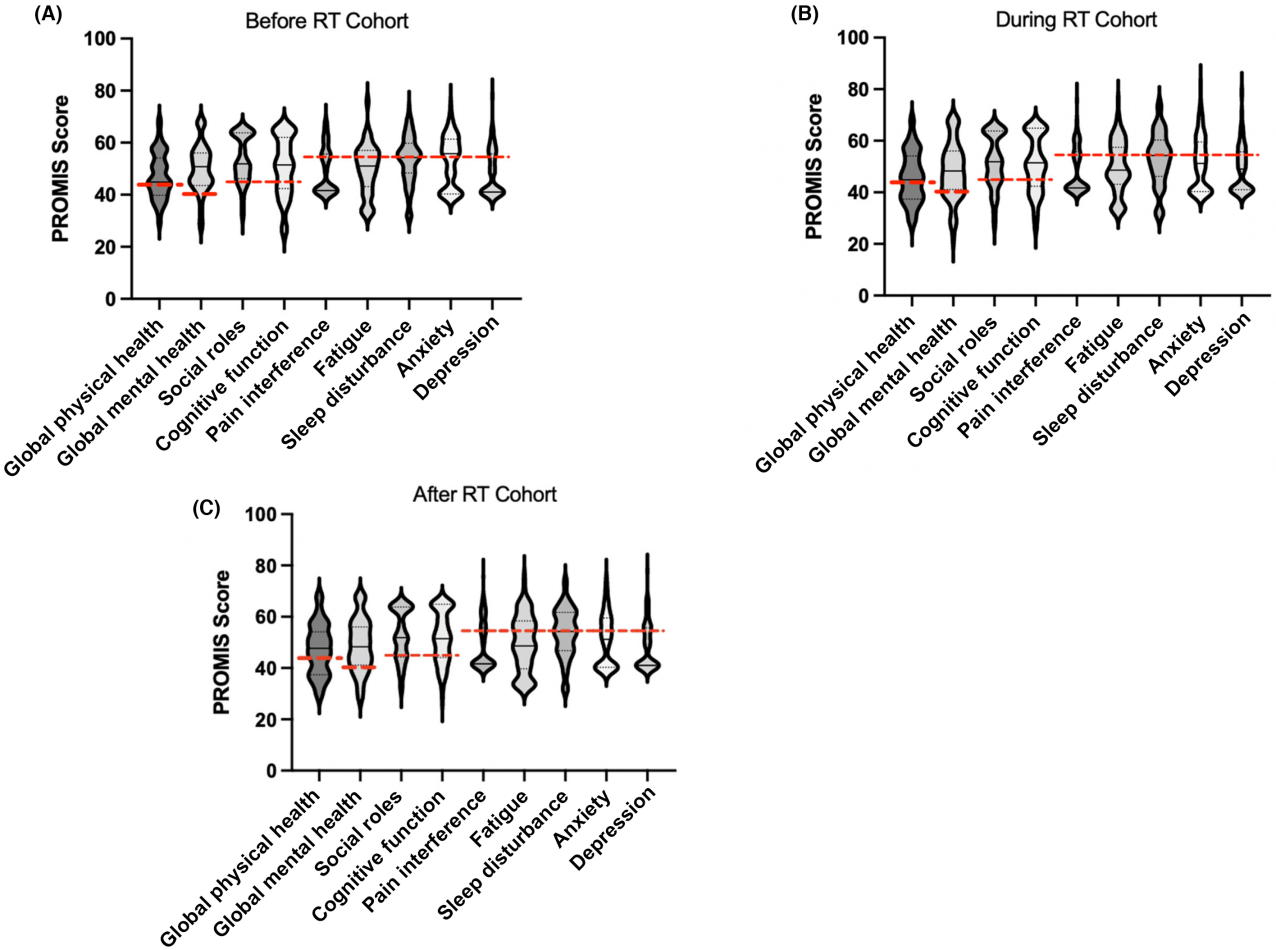
Violin plots to indicate distribution of PROMIS health‐related quality of life scores in the (A) before, (B) during, and (C) after RT cohorts. Higher score indicates better function for global physical health, global mental health, social roles, and cognitive function. Higher score indicates worse function for pain interference, fatigue, sleep disturbance, anxiety, and depression. Black solid line indicates median score. Black dotted lines indicate 25% and 75% quartile scores. Red dotted line indicates the score threshold for mild (social roles [45], cognitive function [45], pain [55], fatigue [55], sleep disturbance [55], anxiety [55], and depression [55]) or fair (global physical health [42], global mental health [40]) or worse severity as established by T‐score distribution maps.^19^ PROMIS, Patient‐Reported Outcomes Measurement Instrument System®.

Eighty‐seven AYAs completed the survey before starting RT (“before RT”). The median (IQR) time between survey completion and starting RT was 2.6 (1.0–3.2) months. Score distributions are shown in Figure 1A. Twenty‐seven percent of AYAs scored as fair or poor severity in the global physical health domain and 11% in the global mental health domain. Twenty‐one percent of AYAs scored as mild distress or worse severity in the social roles domain, 37% in the cognitive function domain, 38% in the pain domain, 40% in the fatigue domain, 51% in the sleep disturbance domain, 60% in the anxiety domain, and 33% in the depression domain. This cohort had a mean anxiety score of 55.2 (SD: 9), which exceeded the MID threshold and signified a difference in score that may cause a change in patient value or care (*p* < 0.001). Thus, there was meaningfully worse anxiety in AYAs before starting RT than in the general US population The remaining mean PROMIS scores were not meaningfully different in magnitude than the general US population, though sleep disturbance was elevated (Table 2).

**TABLE 2:** Mean PROMIS health‐related quality of life scores in adolescents and young adults with cancer in the before, during, and post‐RT cohorts (*n* = 265).

	Before RT (*n* = 87)	During RT^a^ (*n* = 84)	After RT^b^ (*n* = 94)
	Mean score (SD)	Mean score (SD)	Mean score (SD)
Global physical health^c^	48.7 (8.9)	44.9** (9.8)	45.5 (9.5)
Global mental health^c^	50.9 (8.6)	46.2 (10.3)	47.9 (9.6)
Social roles^c^	53.3 (8.6)	51.7 (10.1)	52.9 (9.7)
Cognitive function^c^	49.7 (11.2)	49.7 (10.8)	52.0 (10.0)
Pain interference^d^	50.8 (9.0)	49.3 (8.9)	48.7 (9.2)
Fatigue^d^	51.4 (8.5)	50.3 (10.8)	49.8 (10.7)
Sleep disturbance^d^	54.3 (8.4)	53.8 (9.6)	53.9 (9.3)
Anxiety^d^	55.2** (9.0)	52.6 (10.4)	51.0 (10.0)
Depression^d^	49.2 (8.5)	50.2 (9.3)	49.7 (8.6)

*Note*: Bolded score with ** indicates a meaningful impact from cancer as compared to the general US population.

Abbreviations: PROMIS, Patient‐Reported Outcomes Measurement Instrument System®; RT, radiation therapy; SD, standard deviation.

^a^ Survey completed during RT or within 2 months following completion of RT.

^b^ Survey completed 2 months or greater following completion of RT.

^c^ Higher scores indicate better function.

^d^ Higher scores indicate worse function.

On multivariable linear regression modeling of the before RT cohort, there were no significant relationships between covariates and PROMIS score (Table S2).

### 3.3 During RT cohort

Eighty‐four AYAs completed the survey during RT or within 2 months of RT completion (“during RT”). Score distributions are shown in Figure 1B. Thirty‐seven percent of AYAs scored as fair or poor severity in the global physical health domain and 25% in the global mental health domain. Thirty‐two percent of AYAs scored as mild distress or worse severity in the social roles domain, 35% in the cognitive function domain, 40% in the pain domain, 50% in the fatigue domain, 58% in the sleep disturbance domain, 48% in the anxiety domain, and 38% in the depression domain. This cohort had a mean global physical health score of 44.9 (SD: 9.8), which exceeded the MID threshold and signified a difference in score that may cause a change in patient value or care (*p* < 0.001). Thus, there was meaningfully worse global physical health in AYAs during or shortly after RT than in the general US population. The remaining mean PROMIS scores were not meaningfully different than the general US population; although, sleep disturbance remained elevated (Table 2).

On multivariable linear regression modeling of the during RT cohort, there were no statistically significant relationships between developmental stage, sex, cancer type, or CRT receipt and PROMIS score (Table 3), though patients who received CRT tended to have lower global mental health scores than those who received RT alone (*B* = ‐6.61, standard error [SE] = 3.52, *p* = 0.04). In this model, patients who identified as Hispanic or non‐White race and ethnicity reported decreased fatigue (*B* = ‐18.00, SE = 6.10, *p* < 0.01) compared to patients who identified as non‐Hispanic White race and ethnicity. Patients with regional/distant disease reported worse pain (*B* = 15.94, SE = 5.51, *p* < 0.01) and more fatigue (*B* = 14.20, SE = 5.80, *p* = 0.01) than patients with localized disease.

**TABLE 3:** Linear regression model examining differences in health‐related quality of life scores in adolescents and young adults with cancer in the “during RT” cohort (*n* = 84). Linear regression estimated parameters (*B*, representing the relationship between predictor variables and PROMIS score) were reported with standard error measurements.

	Global physical health^a^		Global mental health^a^		Social roles^a^		Cognitive function^a^		Pain interference^b^		Fatigue^b^		Sleep disturbance^b^		Anxiety^b^		Depression^b^	
	*B*	SE	*B*	SE	*B*	SE	*B*	SE	*B*	SE	*B*	SE	*B*	SE	*B*	SE	*B*	SE
Developmental stage																		
Adolescents versus emerging adults (ref)	−0.58	4.16	3.08	4.22	−3.05	4.26	3.25	4.23	−0.85	3.81	1.34	4.05	−2.28	4.03	−1.41	3.83	−0.68	2.31
Young adults versus emerging adults (ref)	1.92	2.49	0.69	2.92	−0.51	2.87	2.11	2.84	−0.14	2.47	0.78	2.52	−5.07	2.63	−2.21	2.82	1.23	2.51
Systemic therapy																		
Concurrent systemic therapy and RT versus RT alone (ref)	−1.95	3.06	−6.61	3.52	−4.69	3.23	0.15	3.48	1.32	2.77	−1.22	2.85	−2.25	3.39	5.73	3.43	4.27	3.03
Sex																		
Female versus male (ref)	−2.49	2.26	−5.00	2.68	−1.52	2.42	−2.49	2.61	1.39	2.03	4.29	2.13	−0.82	2.53	3.88	2.56	1.09	2.26
Race/ethnicity																		
Non‐white versus white (ref)	8.36	6.39	6.73	7.56	7.75	7.31	6.50	3.87	−11.45	6.45	−18.00**	***6.14***	−2.17	7.67	−7.07	7.75	−10.89	6.86
Cancer stage																		
Regional/distant versus localized (ref)	−6.69	5.80	−3.74	7.07	0.23	6.84	−4.29	3.06	15.94**	***5.51***	14.20**	***5.80***	4.15	6.89	8.46	6.96	4.50	6.16

*Note*: This model also adjusted for cancer type. Bolded score with ** indicates statistically significant result with *p* ≤ 0.01.

Abbreviations: RT, radiation therapy; SE, standard error.

^a^ Higher scores indicate better function.

^b^ Higher scores indicate worse function.

**FIGURE 2:**
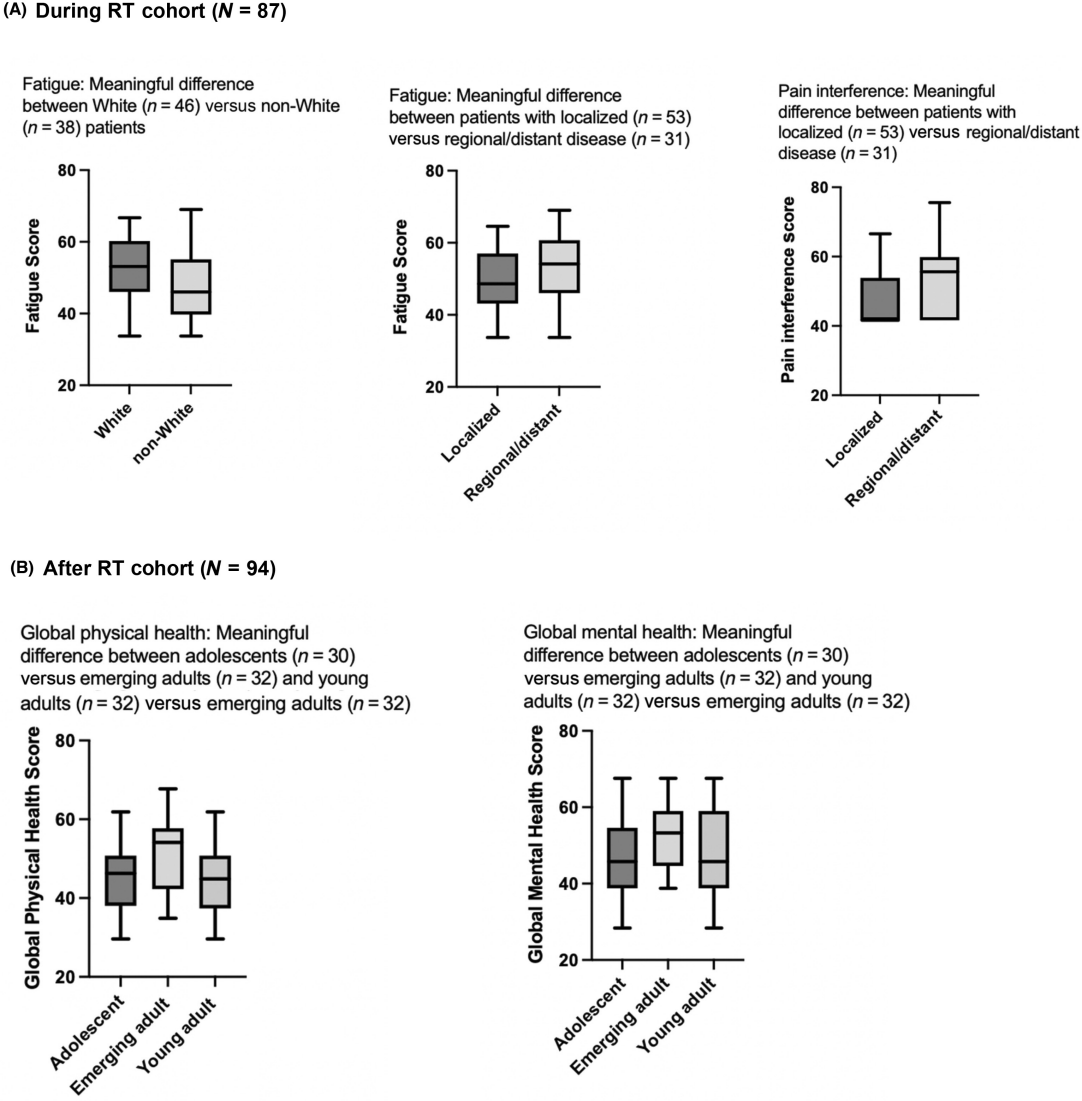
PROMIS health‐related quality of life scores with linear regression significant relationship that demonstrate meaningful clinical impact in the (A) during and (B) after RT cohorts. Black solid line indicates mean score. Higher score indicates better function for global physical health and global mental health. Higher score indicates worse function for fatigue and pain interference. RT, Radiation therapy; PROMIS, Patient‐Reported Outcomes Measurement Instrument System®.

Of the significant relationships found in the linear regression model, the difference in mean scores in the following domains exceeded the MID threshold and were deemed a clinically meaningful difference: fatigue (mean score [SD]: 52.7 [8.3] vs. 47.9 [10.6]) in non‐Hispanic White versus Hispanic and non‐White patients; fatigue (mean score [SD]: 53.8 [9.0] vs. 49.1 [9.5]) and pain (mean score [SD]: 53.3 [9.1] vs. 46.7 [7.9]) in patients with regional/distant disease versus localized disease (Figure 2A).

### 3.4 After RT cohort

Ninety‐four AYAs completed the survey 2 months or greater following RT completion (“after RT”). The median (IQR) time between completing RT and survey completion was 24 (14–27) months. Score distributions are shown in Figure 1C. Thirty‐three percent of AYAs scored as fair or poor severity in the global physical health domain and 22% in the global mental health domain. Twenty‐eight percent of AYAs scored as mild distress or worse severity in the social roles domain, 31% in the cognitive function domain, 36% in the pain domain, 45% in the fatigue domain, 52% in the sleep disturbance domain, 43% in the anxiety domain, and 34% in the depression domain. This cohort had mean PROMIS scores that were not meaningfully different than the general US population; although, sleep disturbance remained elevated (Table 2).

On multivariable linear regression modeling of the after RT cohort, there were no significant relationships between sex, cancer type, cancer stage, or CRT receipt and PROMIS score (Table 4). In this model, adolescents (15–18 years old) reported worse global physical health (*B* = ‐6.87, SE = 2.35, *p* < 0.01) and global mental health (*B* = ‐6.74, SE = 2.16, *p* < 0.01) than emerging adults (19–25 years old). Similarly, young adults (26–39 years old) reported worse global physical health (*B* = ‐7.87, SE = 2.46, *p* < 0.01) and global mental health (*B* = ‐5.67, SE = 2.52, *p* = 0.01) than emerging adults. While not statistically significant, there was a trend toward worse anxiety in the adolescent population (*B* = 4.91, SE = 2.18, *p* = 0.03) and the young adult population (*B* = 4.51, SE = 2.43, *p* = 0.04) as compared to the emerging adult population.

**TABLE 4:** Linear regression model examining differences in health‐related quality of life scores in adolescents and young adults with cancer in the “after RT” cohort (*n* = 94). Linear regression estimated parameters (*B*, representing the relationship between predictor variables and PROMIS score) were reported with standard error measurements.

	Global physical health^a^		Global mental health^a^		Social roles^a^		Cognitive function^a^		Pain interference^b^		Fatigue^b^		Sleep disturbance^b^		Anxiety^b^		Depression^b^	
	*B*	SE	*B*	SE	*B*	SE	*B*	SE	*B*	SE	*B*	SE	*B*	SE	*B*	SE	β	SE
Developmental stage																		
Adolescents versus emerging adults (ref)	−6.87**	***2.35***	−6.74**	***2.16***	−0.62	2.33	−3.04	2.35	−0.29	2.21	2.38	2.51	−2.41	2.26	4.91	2.18	3.64	1.84
Young adults versus emerging adults (ref)	−7.87**	***2.46***	−5.67**	***2.52***	−0.01	2.30	−1.64	2.42	0.56	2.26	2.63	2.55	−1.50	2.33	4.51	2.43	3.18	2.13
Systemic therapy																		
Concurrent systemic therapy and RT versus RT alone (ref)	−0.33	2.40	−2.71	2.33	−4.69	3.23	−2.60	2.39	3.15	2.20	2.44	2.54	4.19	2.12	1.36	2.39	2.29	2.11
Sex																		
Female versus male (ref)	0.91	2.17	3.40	2.14	−1.52	2.42	0.87	2.10	−2.44	1.94	−3.28	1.47	−0.64	1.87	−2.93	2.10	−2.06	1.86
Race/ethnicity																		
Non‐white versus white (ref)	−0.21	1.00	2.40*	1.00	5.75	4.31	−1.82	1.01	−0.89	0.93	−1.88	1.08	−1.90	0.89	−1.16	1.01	−1.05	0.89
Cancer stage																		
Regional/distant versus localized (ref)	4.77	4.93	0.48	4.77	0.23	6.84	−5.89	4.71	1.19	4.36	0.84	5.04	5.10	4.20	1.81	4.73	−4.43	4.18

*Note*: This model also adjusted for cancer type. Bolded score with ** indicates statistically significant result with *p* ≤ 0.01.

Abbreviations: RT, radiation therapy; SE, standard error.

^a^ Higher scores indicate better function.

^b^ Higher scores indicate worse function.

Of the significant relationships found in the linear regression model, the difference in mean scores in the following domains exceeded the MID threshold and were deemed a clinically meaningful difference. In adolescents versus emerging adults: global physical health (mean score [SD]: 45.5 [8.6] vs. 52.3 [10.6]) and global mental health (mean score [SD]: 46.4 [9.0] vs. 53.2 [9.1]). In young adults versus emerging adults: global physical health (mean score [SD]: 44.5 [8.6] vs. 52.3 [10.6]) and global mental health (mean score [SD]: 47.5 [11.3] vs. 53.2 [9.1]) (Figure 2B).

### 3.5 Overall cohort

On multivariable linear regression modeling of all patients, there were no significant relationships among the RT cohort (before vs. during RT vs. after RT) and PROMIS score (Table S3).

## 4 DISCUSSION

This was the first study to investigate self‐reported HRQOL in AYAs with cancer in the before, during, and after RT settings. Before RT, AYAs reported meaningfully worse anxiety as compared to the general US population. During RT, AYAs reported meaningfully worse global physical health. Additionally, we identified specific populations at‐risk for poor short‐term HRQOL while receiving RT. During and shortly after RT, patients with regional/distant disease reported meaningfully worse fatigue and pain than patients with localized disease. Finally, we identified that developmental stage was associated with differing long‐term HRQOL in AYAs after RT completion. The adolescent (15–18 years old) and young adult (26–39 years old) populations reported meaningfully worse global physical health and global mental health than the emerging adult population (19–25 years old).

Our results are consistent with several prior studies showing worse physical outcomes in AYAs undergoing cancer treatment as compared to the general population.^17^, ^19^, ^20^, ^21^ For patients actively receiving RT, worse physical health could be due to the loss of a normal exercise routine, side effects from RT, or from greater radiation dose distributions to organs that affect physical activity, such as the heart or brain.^22^, ^23^, ^24^, ^25^ In addition to the burdensome radiation treatment process, impaired physical health may lead to development of comorbidities, work disruptions, or decreased social engagement, which are all significant contributions to morbidity for AYAs.^26^, ^27^, ^28^, ^29^ This effect has been shown in childhood cancer patients,^30^ but remains understudied in AYAs. Thus, longitudinal HRQOL analyses are needed in AYAs to determine trends in physical health during and after RT so that early interventions can be developed to mitigate potentially detrimental downstream effects.

This study identified advanced cancer stage as a predictor of worse short‐term HRQOL during radiation treatment in AYAs. AYAs with advanced cancer reported experiencing more fatigue and pain than patients with earlier stage disease. Prior studies in cancer patients have shown the prevalence of fatigue and pain in those with advanced disease.^31^, ^32^ These studies have also suggested the adverse effects that these symptoms have on the lifestyle of a cancer patient with advanced disease, including difficulties with activities of daily living for patients with fatigue and increased emergency room visits for patients with pain.^31^, ^33^ Moreover, other studies have shown unique HRQOL needs in palliative settings in AYAs with cancer.^34^ Taken together, these results highlight the need for high‐quality, individualized support services for AYAs with advanced cancer stage to improve their HRQOL.

AYAs with cancer have psychosocial distress during cancer treatment and into survivorship, which has been well established.^17^, ^21^, ^35^, ^36^, ^37^, ^38^, ^39^ Our study showed that this relationship may be especially true in AYAs undergoing CRT as they tended to have worse short‐term global mental health than patients receiving RT without concurrent systemic therapy. Prior studies have corroborated worse HRQOL from multimodality cancer therapy as compared to single modality therapy in the AYA cancer population.^40^ This reveals a specific population that may benefit from close attention or additional specialty referrals, such as psychology and/or psychiatry, during treatment. Future studies should consider an in‐depth examination of adverse events that occur in this population to correlate with patient‐reported HRQOL.

This study also identified developmental stage as a predictor of differing long‐term HRQOL within the AYA population during the post‐RT survivorship period. We found worse global physical health and global mental health, and a trend toward worse anxiety, in AYAs as compared to emerging adults. Each developmental life stage has unique stressors, such as school performance in adolescents, difficulties with employment in emerging adults, and financial hardship in young adults, that contribute to distress during survivorship.^41^, ^42^, ^43^ Subsequently, HRQOL likely varies between each group based on these unique stressors and changes over time as priorities shift. In our study, the differences in HRQOL by developmental stage occurred during the posttreatment survivorship period; there were few differences during the active cancer treatment period. This may signify the overlap in HRQOL during cancer treatment followed by unique HRQOL according to developmental stage after cancer treatment completion and once patients are able to resume their normal lifestyle.

Different radiation treatment characteristics, such as modality, dose/regimen, timing, and intent, may have an impact on toxicities and HRQOL in AYAs with cancer. This study is one of the first to investigate PROs in AYAs receiving RT,^8^ and broadly examined the relationship between RT timepoint and HRQOL. Since radiation characteristics vary significantly by cancer type, future studies should consider analyzing the relationships between radiation characteristics and HRQOL in each cancer type. For example, an analysis of the effect of radiation dose distribution on HRQOL in AYAs by cancer type would be useful to guide cancer type‐specific treatment planning; such an analysis is currently underway. Additionally, an analysis of radiation characteristics in AYAs receiving palliative‐intent RT would be useful to determine the value of RT in end‐of‐life settings. Thus, many opportunities exist for future studies in AYAs with cancer receiving RT.

This study has some limitations. First, the study design was cross‐sectional and did not analyze PRO responses longitudinally, which limited our comparison of scores among the three independent RT cohorts. Additionally, our patient population was limited in sample size, heterogeneous between cohorts, and consisted of many different cancer types. Our analysis of each cancer type was limited due to sample size. Our analysis of mean HRQOL scores was also limited due to lack of a control group or of available data in the literature regarding similar scores in older adults with cancer undergoing RT. However, it is still important to show that AYAs with cancer have worse HRQOL before and during RT as compared to the general population to demonstrate the need for improved resources in this population. While our survey captured a comprehensive set of physical, mental, and social HRQOL domains, it was lacking some HRQOL domains particularly relevant to AYAs, such as fertility, financial hardship, and body image, and it did not capture patient‐reported adverse event data. Future studies should consider analyzing AYA‐relevant HRQOL domains and patient‐reported adverse events to further characterize AYA well‐being during RT. Despite the limitations of this retrospective, cross‐sectional analysis, this study serves as a benchmark for future studies to further investigate HRQOL in the AYA RT population.

## 5 CONCLUSIONS

This study was the first to investigate patient‐reported HRQOL in AYAs with cancer in the before, during, and after RT settings. We found that AYAs who received RT are at‐risk for impaired HRQOL during and after treatment, and we identified several demographic and clinical characteristics associated with this increased risk. Our results highlight unique insight into the need to improve HRQOL in AYAs during RT and contribute to the accumulating literature demonstrating the need to improve HRQOL in this population during survivorship.

## AUTHOR CONTRIBUTIONS

**Kelsey L Corrigan:** Conceptualization (lead); data curation (lead); formal analysis (lead); investigation (lead); writing – original draft (lead). **Bryce B. Reeve:** Formal analysis (equal); methodology (equal); writing – review and editing (equal). **John M. Salsman:** Formal analysis (supporting); investigation (supporting); validation (supporting); writing – review and editing (supporting). **Elizabeth J Siembida:** Formal analysis (supporting); methodology (equal); writing – review and editing (equal). **Grace L Smith:** Conceptualization (equal); methodology (equal); resources (equal); supervision (equal); visualization (equal); writing – review and editing (equal). **Maria Swartz:** Formal analysis (equal); writing – review and editing (equal). **Kamaria Lee:** Data curation (equal); writing – review and editing (equal). **Faraz Afridi:** Data curation (equal); writing – review and editing (equal). **Lauren M Andring:** Data curation (equal); formal analysis (equal); writing – review and editing (equal). **Andrew J. Bishop:** Conceptualization (equal); methodology (equal); writing – review and editing (equal). **Jillian Gunther:** Conceptualization (equal); writing – review and editing (equal). **J Andrew Livingston:** Data curation (supporting); resources (supporting); writing – review and editing (equal). **Susan Peterson:** Writing – review and editing (equal). **Michael E Roth:** Conceptualization (equal); investigation (equal); methodology (equal); project administration (equal); resources (equal); supervision (equal); writing – original draft (supporting); writing – review and editing (equal).

## FUNDING INFORMATION

The work was supported by NIH Cancer Center Support Grant P30 CA016672.

## CONFLICT OF INTEREST STATEMENT

The authors report no financial disclosures or conflicts of interests related to this work.

## Supporting information


Table S1:
Table S2:
Table S3:

## Data Availability

Research data are stored in an institutional repository and will be shared upon request to the corresponding author.
