# Supplementary material for: Patient‐reported quality of life in adolescents and young adults with cancer who received radiation therapy
Source: Cancer Med. 2023 May 18;12(13):14157–70. doi: 10.1002/cam4.6082 (PMC10358202; doi:10.1002/cam4.6082)
Supplement: Supplementary file 1 — Table S1: Table S2: Table S3: [file CAM4-12-14157-s001.docx]

**SUPPLEMENTAL DATA**

**Supplemental Table 1:** Histopathologic designation for each cancer type among cohorts (N=265).

|  | **Before RT (n=87)**  **N (%)** | **During RT^a^ (n=84)**  **N (%)** | **After RT^b^ (n=94)**  **N (%)** | **Total (n=265)**  **N (%)** |
| --- | --- | --- | --- | --- |
| Breast cancer |  |  |  |  |
| DCIS | 1 (1.1%) | 0 | 0 | 1 (0.4%) |
| Invasive ductal carcinoma | 11 (13.8%) | 6 (7.1%) | 7 (7.4%) | 24 (9.1%) |
| Central nervous system cancer |  |  |  |  |
| Astrocytoma | 7 (8.0%) | 7 (8.3%) | 7 (7.4%) | 21 (7.9%) |
| Brainstem glioma | 0 | 1 (1.2%) | 0 | 1 (0.4%) |
| Choroid plexus carcinoma | 1 (1.1%) | 0 | 0 | 1 (0.4%) |
| Ependymoma | 0 | 0 | 1 (1.2%) | 1 (0.4%) |
| Germ cell tumor | 0 | 3 (3.6%) | 0 | 3 (1.1%) |
| Glioblastoma | 4 (4.6%) | 8 (9.5%) | 9 (9.6%) | 21 (7.9%) |
| Medulloblastoma | 0 | 2 (2.4%) | 0 | 2 (0.8%) |
| Meningioma | 0 | 1 (1.2%) | 0 | 1 (0.4%) |
| Oligodendroglioma | 2 (2.3%) | 3 (3.6%) | 2 (2.1%) | 7 (2.6%) |
| Pineoblastoma | 0 | 1 (1.2%) | 0 | 1 (0.4%) |
| Gastrointestinal cancer |  |  |  |  |
| Adenocarcinoma (colorectal) | 4 (4.6%) | 2 (2.4%) | 2 (2.1%) | 8 (3.0%) |
| Adenocarcinoma (pancreas) | 0 | 0 | 1 (1.2%) | 1 (0.4%) |
| Adenocarcinoma (stomach) | 1 (1.1%) | 0 | 0 | 1 (0.4%) |
| Cholangiocarcinoma | 0 | 1 (1.2%) | 0 | 1 (0.4%) |
| Genitourinary cancer |  |  |  |  |
| Renal cell carcinoma | 0 | 1 (1.2%) | 0 | 1 (0.4%) |
| Seminoma | 1 (1.1%) | 0 | 2 (2.1%) | 3 (1.1%) |
| Gynecologic |  |  |  |  |
| Squamous cell carcinoma (cervix) | 1 (1.1%) | 0 (0.0%) | 2 (2.1%) | 3 (1.1%) |
| Head and neck cancer |  |  |  |  |
| Adenoid cystic carcinoma | 1 (1.1%) | 0 | 3 (3.2%) | 4 (1.5%) |
| Nasopharyngeal carcinoma | 0 | 2 (2.4%) | 2 (2.1%) | 4 (1.5%) |
| Sinonasal undifferentiated carcinoma | 1 (1.1%) | 1 (1.2%) | 3 (3.2%) | 4 (1.5%) |
| Squamous cell carcinoma (larynx) | 0 | 1 (1.2%) | 2 (2.1%) | 3 (1.1%) |
| Squamous cell carcinoma (oral cavity) | 2 (2.3%) | 4 (4.8%) | 3 (3.2%) | 9 (3.4%) |
| Squamous cell carcinoma (oropharynx) | 1 (1.1%) | 0 | 0 | 1 (0.4%) |
| Medullary thyroid carcinoma | 0 | 1 (1.2%) | 1 (1.2%) | 2 (0.8%) |
| Leukemia |  |  |  |  |
| ALL | 3 (3.4%) | 3 (3.6%) | 3 (3.2%) | 9 (3.4%) |
| AML | 0 | 1 (1.2%) | 1 (1.2%) | 2 (0.8%) |
| Lymphoma |  |  |  |  |
| Hodgkin’s Lymphoma | 6 (6.9%) | 2 (2.4%) | 6 (9.6%) | 14 (5.3%) |
| Diffuse large B-cell lymphoma | 8 (9.2%) | 3 (3.6%) | 5 (5.3%) | 16 (6.0%) |
| Lymphoblastic lymphoma | 1 (1.1%) | 1 (1.2%) | 0 | 2 (0.8%) |
| NK/T-cell lymphoma | 0 | 1 (1.2%) | 0 | 1 (0.4%) |
| Peripheral T-cell lymphoma | 1 (1.1%) | 0 | 1 (1.2%) | 2 (0.8%) |
| Plasmacytoma | 1 (1.1%) | 0 | 1 (1.2%) | 2 (0.8%) |
| Primary mediastinal B-cell lymphoma | 0 | 0 | 2 (2.1%) | 2 (0.8%) |
| Lung cancer |  |  |  |  |
| Adenocarcinoma | 1 (1.1%) | 4 (4.8%) | 2 (2.1%) | 7 (2.6%) |
| Melanoma/skin cancer |  |  |  |  |
| Melanoma (head and neck) | 2 (2.3%) | 1 (1.2%) | 2 (2.1%) | 5 (1.9%) |
| Melanoma (extremity) | 0 | 1 (1.2%) | 0 | 1 (0.4%) |
| Melanoma (thorax) | 2 (2.3%) | 0 | 0 | 2 (0.8%) |
| Squamous cell carcinoma (extremity) | 0 | 1 (1.2%) | 0 | 1 (0.4%) |
| Soft tissue sarcoma |  |  |  |  |
| Alveolar soft part sarcoma | 1 (1.1%) | 0 | 1 (1.2%) | 2 (0.8%) |
| Angiosarcoma | 1 (1.1%) | 1 (1.2%) | 0 | 2 (0.8%) |
| Chondrosarcoma | 0 | 1 (1.2%) | 1 (1.2%) | 2 (0.8%) |
| Chordoma | 0 | 0 | 1 (1.2%) | 1 (0.4%) |
| Clear cell sarcoma | 2 (2.3%) | 1 (1.2%) | 1 (1.2%) |  |
| Desmoid fibromatosis | 0 | 0 | 1 (1.2%) | 1 (0.4%) |
| Desmoplastic round cell carcinoma | 1 (1.1%) | 0 | 0 | 1 (0.4%) |
| Epithelioid sarcoma | 3 (3.4%) | 3 (3.6%) | 2 (2.1%) | 8 (3.0%) |
| Ewing sarcoma | 4 (4.6%) | 1 (1.2%) | 3 (3.2%) | 8 (3.0%) |
| Fibromyxoid sarcoma | 0 | 1 (1.2%) | 0 | 1 (0.4%) |
| Hemangiopericytoma | 1 (1.1%) | 0 | 0 | 1 (0.4%) |
| Leiomyosarcoma | 2 (2.3%) | 0 | 1 (1.2%) | 3 (1.1%) |
| Liposarcoma | 0 | 0 | 3 (3.2%) | 3 (1.1%) |
| MPNST | 1 (1.1%) | 1 (1.2%) | 1 (1.2%) | 3 (1.1%) |
| Myxoid liposarcoma | 4 (4.6%) | 2 (2.4%) | 2 (2.1%) | 8 (3.0%) |
| Osteosarcoma | 0 | 2 (2.4%) | 2 (2.1%) | 4 (1.5%) |
| Rhabdomyosarcoma | 2 (2.3%) | 3 (3.6%) | 1 (1.2%) | 6 (2.3%) |
| Solitary fibrous tumor | 0 | 1 (1.2%) | 0 | 1 (0.4%) |
| Spindle cell sarcoma | 0 | 2 (2.4%) | 2 (2.1%) | 4 (1.5%) |
| Synovial sarcoma | 1 (1.1%) | 2 (2.4%) | 2 (2.1%) | 5 (1.9%) |

**Supplemental Table 2:** Linear regression model examining differences in health-related quality of life scores in adolescents and young adults with cancer in the ‘before RT’ cohort (n=87). Linear regression estimated parameters (*B,* representing the relationship between predictor variables and PROMIS score) were reported with standard error measurements. No significant relationships (defined as p-value ≤0.01) were identified. Abbreviations: Radiation therapy (RT); Standard error (SE).

|  | Global Physical Health^a^ | | Global Mental Health^a^ | | Social Roles^a^ | | Cognitive Function^a^ | | Pain Interference^b^ | | Fatigue^b^ | | Sleep Disturbance^b^ | | Anxiety^b^ | | Depression^b^ | |
| --- | --- | --- | --- | --- | --- | --- | --- | --- | --- | --- | --- | --- | --- | --- | --- | --- | --- | --- |
|  | *B* | SE | *B* | SE | *B* | SE | *B* | SE | *B* | SE | *B* | SE | *B* | SE | *B* | SE | *B* | SE |
| Developmental stage |  |  |  |  |  |  |  |  |  |  |  |  |  |  |  |  |  |  |
| Adolescents vs. emerging adults (ref) | -1.70 | 2.49 | -0.43 | 2.87 | 1.57 | 3.04 | 1.61 | 3.01 | 3.63 | 3.22 | 1.01 | 2.64 | 1.43 | 3.01 | 2.08 | 3.08 | 3.41 | 2.79 |
| Young adults vs. emerging adults (ref) | 1.24 | 1.79 | 0.79 | 2.33 | 0.16 | 2.63 | 1.57 | 3.11 | 0.93 | 2.54 | 0.99 | 2.52 | 3.34 | 2.48 | -1.35 | 2.65 | -1.21 | 2.42 |
| Systemic therapy |  |  |  |  |  |  |  |  |  |  |  |  |  |  |  |  |  |  |
| Concurrent systemic therapy and RT vs. RT alone (ref) | -4.52 | 3.81 | -2.10 | 4.02 | -2.39 | 4.26 | 3.29 | 5.45 | 4.02 | 4.24 | -1.42 | 4.07 | 4.48 | 4.05 | -0.37 | 4.57 | 2.10 | 4.18 |
| Sex |  |  |  |  |  |  |  |  |  |  |  |  |  |  |  |  |  |  |
| Female vs. Male (ref) | -0.51 | 2.39 | 3.22 | 2.52 | -2.82 | 2.66 | -0.89 | 3.42 | 0.52 | 2.75 | 2.10 | 2.56 | -1.19 | 2.54 | -1.35 | 2.88 | -0.02 | 2.63 |
| Race/ethnicity |  |  |  |  |  |  |  |  |  |  |  |  |  |  |  |  |  |  |
| Non-White vs. White (ref) | -6.78 | 5.78 | -2.04 | 6.11 | 2.93 | 6.39 | 5.40 | 8.28 | 9.91 | 6.46 | 7.10 | 6.14 | 6.09 | 6.14 | 1.36 | 6.91 | -0.07 | 6.31 |
| Cancer stage |  |  |  |  |  |  |  |  |  |  |  |  |  |  |  |  |  |  |
| Regional/distant vs. Localized (ref) | -9.40 | 4.86 | 3.82 | 5.13 | 8.81 | 5.39 | 9.24 | 6.97 | -6.64 | 5.50 | -6.15 | 5.16 | -1.74 | 5.16 | -3.81 | 5.80 | -5.99 | 5.31 |
| ^a^ Higher scores indicate better function.  ^b^ Higher scores indicate worse function.  Note: This model also adjusted for cancer type. | | | | | | | | | | | | | | | | | | |

**Supplementary Table 3:** Linear regression model examining differences in health-related quality of life scores in adolescents and young adults with cancer in all three RT cohorts (n=265). Linear regression estimated parameters (*B,* representing the relationship between predictor variables and PROMIS score) were reported with standard error measurements. No significant relationships (defined as p-value ≤0.01) were identified. Abbreviations: Radiation therapy (RT); Standard error (SE).

|  | Global Physical Health^a^ | | Global Mental Health^a^ | | Social  Roles^a^ | | Cognitive Function^a^ | | Pain Interference^b^ | | Fatigue^b^ | | Sleep Disturbance^b^ | | Anxiety^b^ | | Depression^b^ | |
| --- | --- | --- | --- | --- | --- | --- | --- | --- | --- | --- | --- | --- | --- | --- | --- | --- | --- | --- |
|  | *B* | SE | *B* | SE | *B* | SE | *B* | SE | *B* | SE | *B* | SE | *B* | SE | *B* | SE | *B* | SE |
| RT Cohort |  |  |  |  |  |  |  |  |  |  |  |  |  |  |  |  |  |  |
| During RT vs. before RT (ref) | -1.49 | 1.54 | -1.48 | 1.67 | -1.62 | 1.67 | -0.01 | 1.89 | -1.67 | 1.55 | -0.57 | 1.56 | -0.95 | 1.58 | -2.77 | 1.69 | 1.01 | 1.53 |
| After RT vs. before RT (ref) | -1.47 | 1.49 | -0.17 | 0.75 | -0.18 | 0.75 | 1.23 | 0.84 | -1.07 | 0.73 | -0.84 | 0.80 | -0.24 | 0.72 | -2.12 | 0.77 | -0.42 | 0.69 |
| Developmental stage |  |  |  |  |  |  |  |  |  |  |  |  |  |  |  |  |  |  |
| Adolescents vs. emerging adults (ref) | -3.69 | 1.74 | -3.15 | 1.73 | -0.88 | 1.78 | -0.43 | 1.82 | 0.21 | 1.73 | 1.42 | 1.78 | -2.15 | 1.68 | 2.54 | 1.69 | 1.90 | 1.43 |
| Young adults vs. emerging adults (ref) | -1.90 | 1.44 | -2.42 | 1.50 | 0.08 | 1.44 | -1.06 | 1.61 | 0.14 | 1.36 | 1.44 | 1.44 | -2.87 | 1.39 | 2.61 | 1.51 | 1.70 | 1.35 |
| Systemic therapy |  |  |  |  |  |  |  |  |  |  |  |  |  |  |  |  |  |  |
| Concurrent systemic therapy and RT vs. RT alone (ref) | -1.49 | 1.62 | -3.63 | 1.68 | -1.97 | 1.63 | -1.23 | 1.81 | 2.98 | 1.53 | 0.84 | 1.68 | 2.40 | 1.58 | 2.71 | 1.68 | 2.43 | 1.51 |
| Cancer stage |  |  |  |  |  |  |  |  |  |  |  |  |  |  |  |  |  |  |
| Regional/distant vs. Localized (ref) | 0.81 | 1.31 | 0.42 | 1.37 | -0.39 | 1.33 | -0.98 | 1.47 | 2.28 | 1.24 | 0.18 | 1.36 | -0.78 | 1.28 | -1.30 | 1.36 | -0.79 | 1.22 |
| ^a^ Higher scores indicate better function.  ^b^ Higher scores indicate worse function.  Note: This model also adjusted for sex, race/ethnicity, and cancer type. | | | | | | | | | | | | | | | | | | |
